# The Hoffmann Reflex

**DOI:** 10.3390/neurosci7030072

**Published:** 2026-06-17

**Authors:** Oscar Arias-Carrión, Emmanuel Ortega-Robles

**Affiliations:** 1 División de Neurociencias, Clínica, Instituto Nacional de Rehabilitación Luis Guillermo Ibarra Ibarra, Mexico City 14389, Mexico; 2 Tecnologico de Monterrey, Escuela de Medicina y Ciencias de la Salud, Mexico City 14380, Mexico

**Keywords:** Hoffmann reflex, spinal inhibition, sensorimotor integration, rate-dependent depression, translational neurophysiology, precision neurology

## Abstract

The human spinal cord is increasingly recognized as an active and adaptable component of sensorimotor function, contributing to motor control, pain modulation, and recovery after neurological injury. Within this framework, the Hoffmann reflex (H-reflex) has evolved from a classical electrophysiological phenomenon into a useful probe of spinal circuit function. Rather than reflecting motoneuron excitability alone, H-reflex amplitude and modulation arise from the interaction of Ia afferent transmission, presynaptic inhibition, homosynaptic depression, and interneuronal networks that regulate sensorimotor gain in a state-dependent manner. This review synthesizes classical and contemporary evidence to position the H-reflex as an indirect measure of spinal inhibitory function in humans. We integrate physiological mechanisms with findings from studies in chronic pain syndromes, spasticity, Parkinson’s disease, and recovery after central nervous system injury, where alterations in spinal inhibitory processes have been described. We further discuss methodological and conceptual challenges that limit clinical translation, including state dependence, protocol heterogeneity, and the lack of normative reference frameworks. Finally, we outline directions for integrating H-reflex paradigms with complementary approaches to improve the interpretation of spinal circuit function and its relation to clinical phenomena. Framed in this context, the H-reflex can be considered a valuable experimental and translational tool, whose utility depends on careful methodological implementation and physiologically informed interpretation.

## 1. Introduction

Understanding how the human spinal cord regulates sensorimotor gain remains a central challenge in clinical neurology. Although supraspinal mechanisms have traditionally dominated explanatory models of movement disorders, pain syndromes, and recovery after neurological injury, it has become increasingly clear that the spinal cord is not a passive relay but an adaptive, plastic, and highly regulated processing system [1,2]. Failures of spinal inhibitory control contribute importantly to pathological motor output, abnormal sensory amplification, and maladaptive plasticity across a wide spectrum of neurological conditions [3,4].

Among available neurophysiological tools, the Hoffmann reflex (H-reflex) occupies a unique position. Unlike peripheral nerve conduction studies, which primarily assess axonal integrity, or transcranial magnetic stimulation, which evaluates corticospinal excitability, the H-reflex provides a non-invasive and indirect functional measure of spinal sensorimotor circuits in humans [5,6]. By probing the efficacy and modulation of Ia afferent transmission onto α-motoneurons, the H-reflex reflects the combined influence of excitatory synapses, presynaptic inhibitory mechanisms, homosynaptic depression, and multiple classes of inhibitory interneurons [7,8]. As such, it offers a useful window into spinal circuit dynamics that are otherwise difficult to assess in vivo.

**Figure 1:**
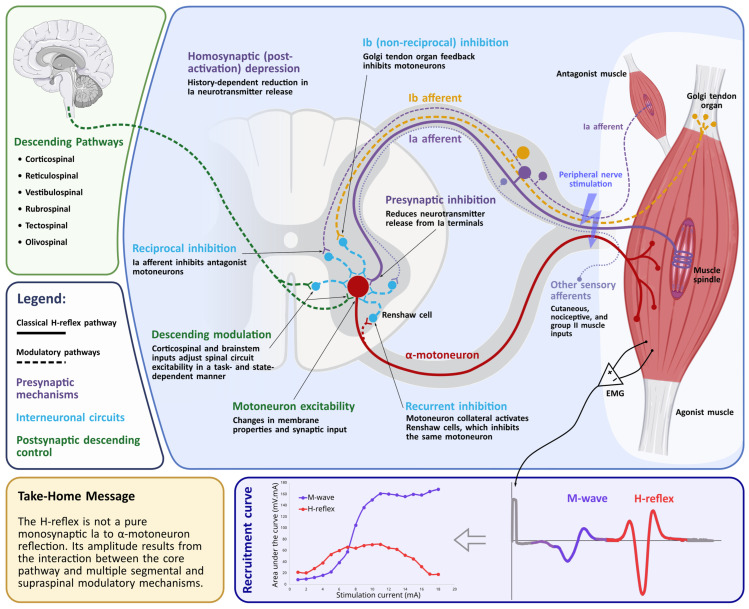
Circuit-level determinants of the H-reflex. Electrical stimulation of peripheral nerves recruits Ia afferents from muscle spindles, which monosynaptically activate α-motoneurons to generate the H-reflex. The final response reflects the integration of multiple modulatory influences, including presynaptic inhibition and homosynaptic (post-activation) depression at Ia terminals, spinal interneuronal circuits (reciprocal Ia, Ib non-reciprocal, and recurrent Renshaw inhibition), other sensory afferents, and descending supraspinal inputs that dynamically regulate motoneuron excitability. For schematic clarity, other sensory afferents are grouped together and include cutaneous, nociceptive, and group II muscle inputs, which may modulate spinal excitability primarily through polysynaptic interneuronal pathways in a task- and context-dependent manner. The lower panel illustrates the M-wave, generated by direct activation of motor axons, and the H-reflex, along with their characteristic recruitment curves as stimulation intensity increases.

Historically, the H-reflex was interpreted largely as a surrogate of motoneuron pool excitability. This interpretation is now considered incomplete. Experimental and clinical evidence accumulated over the past three decades demonstrates that H-reflex amplitude and modulation are shaped to a large extent by presynaptic and interneuronal mechanisms rather than by motoneuron excitability alone [9,10]. Rate-dependent depression, presynaptic inhibition, reciprocal inhibition, Ib-mediated pathways, and recurrent inhibition each contribute in distinct, state-dependent ways, highlighting the sensitivity of the H-reflex to multiple interacting spinal mechanisms rather than a single process [7,11,12]. This conceptual shift has transformed the H-reflex from a descriptive electrophysiological phenomenon into a mechanistically informative probe (Figure 1).

Importantly, interpreting the H-reflex as a probe of spinal inhibitory function does not imply that α-motoneuron excitability is irrelevant. Spinal inhibitory mechanisms ultimately influence motor output by regulating the excitability state and discharge probability of the α-motoneuron pool, which represents the final common pathway of the reflex response. Therefore, abnormal H-reflex excitability, as observed in conditions such as post-stroke spasticity, may reflect impaired inhibitory modulation of Ia afferent input, altered interneuronal control, changes in descending drive, intrinsic motoneuron properties, or their combined effects. Accordingly, conventional H-reflex amplitude measures should be interpreted as net reflex outputs, whereas dynamic or conditioning paradigms are needed to infer the contribution of specific inhibitory mechanisms.

Concurrently, clinical and translational neurophysiology increasingly require biomarkers capable of linking pathophysiological mechanisms with therapeutic response, including responses to pharmacological treatments, physiotherapy and rehabilitation programs, neuromodulatory interventions, and recovery after neurological injury. In chronic pain syndromes, spasticity, Parkinson’s disease, and spinal cord injury, symptoms frequently diverge from structural damage or conventional electrophysiological measures [13,14]. These dissociations have highlighted the importance of functional circuit-level abnormalities—particularly spinal disinhibition—as drivers of disease expression [15,16]. The H-reflex, especially when analyzed using dynamic paradigms such as rate-dependent depression, has emerged as a potentially reproducible marker of disinhibition, with sensitivity to pharmacological and rehabilitative interventions reported in several contexts [17,18,19,20,21].

Recent work has further expanded the translational relevance of H-reflex methodologies. Optimized stimulation protocols, reduced acquisition paradigms, and improved normalization strategies have enhanced feasibility in both clinical and experimental settings [5,22,23,24]. At the same time, converging evidence from animal models and human studies has linked H-reflex abnormalities to specific physiological and, in some cases, molecularly informed mechanisms, including impaired neurotransmitter release at Ia terminals, altered GABAergic control, and maladaptive spinal plasticity [2,7,25]. These advances position H-reflex paradigms not merely as physiological readouts, but as candidate tools for identifying spinal circuit dysfunction and potentially contributing to mechanism-based stratification and precision neurology.

Despite these advances, the field remains fragmented. Methodological heterogeneity, inconsistent terminology, and lingering conceptual ambiguities continue to limit clinical adoption [1,5]. Moreover, the spinal cord is still underrepresented in integrative models of neurological disease, often relegated to a secondary role behind cortical and subcortical structures. A unified, mechanism-oriented framework is therefore needed to clarify how H-reflex-derived measures relate to specific spinal pathways, how they change across awake behavioral and task-related contexts, such as rest, voluntary contraction, posture, locomotion, attention, and training-dependent plasticity, and how they can be leveraged to inform diagnosis, prognosis, and treatment selection.

In this context, the present work synthesizes classical and contemporary evidence to reposition the H-reflex as a key tool for studying human spinal neurophysiology. Throughout the review, we distinguish between the neurophysiological definition of the H-reflex and its mechanism-oriented interpretation. The former refers to the evoked electromyographic response produced by peripheral nerve stimulation through the activation of Ia afferent pathways and subsequent recruitment of α-motoneurons. The latter refers to the use of specific H-reflex paradigms and parameters to infer the contribution of spinal modulatory processes to the final reflex output. By integrating these mechanism-oriented interpretations with quantitative parameters and translational implications, this review aims to clarify the neurobiological substrates underlying H-reflex modulation, delineate its relevance across neurological disorders, and identify critical gaps and challenges that must be addressed to advance the field. Framed in this way, the H-reflex is not only a legacy technique, but a method with continued and evolving relevance for understanding and studying disorders of human sensorimotor control.

## 2. The H-Reflex as a Dynamic Probe of Spinal Sensorimotor Integration

The H-reflex is best understood not as a simple monosynaptic reflex analog, but as a state-dependent measure that reflects multiple interacting mechanisms within spinal sensorimotor integration. Electrical stimulation of a mixed peripheral nerve activates both efferent motor axons and large-diameter Ia afferent fibers arising from the muscle spindle. Direct activation of motor axons generates the short-latency M-wave, recorded by surface electromyography from the target muscle, whereas activation of Ia afferents conveys a sensory volley centrally toward the spinal cord, where these fibers establish monosynaptic excitatory connections with α-motoneurons innervating the same muscle. The resulting reflex discharge travels back along motor axons and produces the H-reflex, a later and typically smaller EMG response. Thus, stimulation above motor threshold evokes two temporally and mechanistically distinct responses in the homonymous muscle. As stimulus intensity increases, the M-wave grows due to progressive recruitment of motor axons, whereas the H-reflex typically increases at low intensities and then decreases at higher intensities due to antidromic collision in motor fibers and the engagement of spinal inhibitory mechanisms. At supramaximal intensities, the H-reflex is abolished due to collision between antidromic motor volleys and orthodromic reflex activity, whereas the M-wave reaches its maximal amplitude, a property that provides an internal physiological reference for stimulus normalization [1,5].

The recorded H-reflex also requires intact neuromuscular transmission. After the reflex motor discharge travels along α-motoneuron axons, acetylcholine released from motor nerve terminals activates nicotinic acetylcholine receptors at the neuromuscular junction, generating muscle fiber depolarization and the electromyographic response detected as the H-reflex [23]. The same final neuromuscular transmission step is required for the M-wave, which is generated by direct activation of motor axons [22]. Therefore, under stable recording conditions and with appropriate M-wave monitoring, H-reflex modulation is generally interpreted as reflecting changes in reflex pathway function rather than primary changes in neuromuscular junction transmission [26].

In this context, the H-reflex and M-wave reflect different aspects of motoneuron pool activation. In accordance with the size principle, α-motoneurons are recruited from smaller, more excitable units innervating slow-twitch muscle fibers to larger units associated with fast motor fibers [27]. Because Ia afferent input produces disproportionately larger excitatory postsynaptic potentials (EPSPs) in small motoneurons, these units tend to contribute more prominently to the H-reflex response. By contrast, electrical stimulation underlying the M-wave preferentially activates large-diameter motor axons, recruiting fast motor units early and bypassing synaptic integration. As a result, the H-reflex does not provide a global estimate of motoneuron output but instead samples a subset of the motoneuron pool, whose composition varies with the awake motor and postural context in which the reflex is elicited, as well as with spinal inhibitory tone (Figure 1) [9,28].

Interpretation also requires attention to differences between extensor and flexor muscles, forelimb/upper-limb and hindlimb/lower-limb muscles, and specific agonist–antagonist pairs [23,29]. Muscles with strong postural or antigravity functions, such as the soleus, usually show more robust H-reflex responses, whereas flexor muscles and many upper-limb muscles may depend more strongly on voluntary facilitation and descending control [30,31]. In addition, reciprocal inhibition, Ib-mediated pathways, recurrent inhibition, and other sensory inputs are organized according to the functional demands of each muscle pair and limb segment [5,32]. Thus, H-reflex findings should not be generalized across muscles without considering the tested muscle, its antagonist, the limb segment, and the motor or task-related context [9,23].

### 2.1. Recruitment Dynamics and Reflex Gain

**Figure 2:**
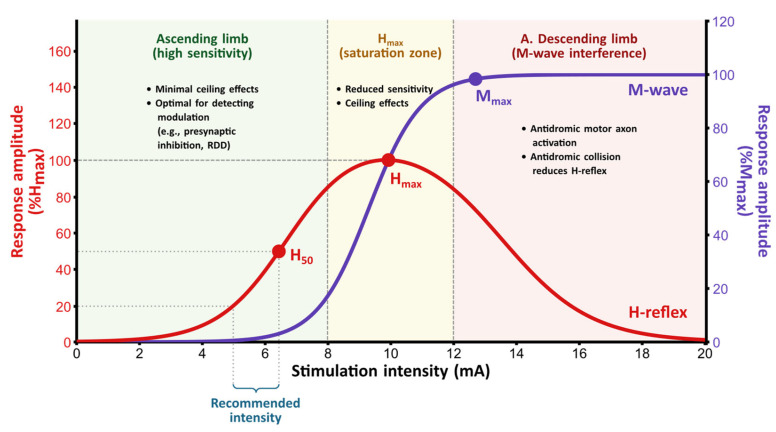
H-reflex and M-wave recruitment curves and optimal sampling regions. The H-reflex (red) and M-wave (purple) exhibit distinct recruitment profiles as stimulation intensity increases. The H-reflex rises along the ascending limb, reaches a peak (H_max_), and declines at higher intensities due to antidromic motor axon activation and collision, whereas the M-wave increases sigmoidally toward M_max_. Sampling at intermediate intensities on the ascending limb (e.g., H_50_) is recommended to optimize sensitivity to physiological changes, including presynaptic inhibition and rate-dependent depression. This schematic representation is intended for conceptual illustration; the relative amplitudes of the H-reflex and M-wave are not depicted to scale.

The relationship between stimulus intensity and H-reflex amplitude typically follows a characteristic sigmoidal recruitment function. Two parameters derived from this curve are of particular physiological relevance: the maximal reflex amplitude (H_max_), reflecting the maximum observable response under specific experimental conditions, and the stimulus intensity required to elicit half-maximal response (H_50_), which is interpreted as an index of the sensitivity of the reflex pathway to afferent input [33]. Together, these parameters are commonly used to characterize the static reflex gain, describing the input–output transformation of the spinal sensorimotor loop at a given moment (Figure 2).

Beyond static measures, reflex gain can be operationalized dynamically as the slope of the relationship between background electromyographic activity and H-reflex amplitude during voluntary contraction [34,35]. This activity-dependent reflex gain reflects the proportion of motoneurons operating within a subliminal fringe—a population considered to be near discharge threshold, whose size expands or contracts under descending drive and sensory feedback [36]. Accordingly, changes in reflex amplitude during movement are thought to reflect, at least in part, shifts in this fringe rather than changes in intrinsic motoneuron excitability per se.

### 2.2. Temporal Integration and Polysynaptic Convergence

Although traditionally described as monosynaptic, the H-reflex primarily reflects monosynaptic Ia transmission but is influenced by a temporally permissive integration window that allows oligosynaptic inputs to modulate motoneuron discharge. In human soleus motoneurons, the relatively slow rise time of composite EPSPs may permit convergent inputs from Ib afferents, recurrent inhibitory circuits, and propriospinal interneurons to influence reflex amplitude [37,38]. These influences become particularly apparent under certain experimental conditions, including when the H-reflex is elicited on the descending limb of the recruitment curve, where slow motoneurons dominate and sensitivity to inhibition and facilitation is thought to differ [39].

Because H-reflex amplitude reflects both Ia transmission and convergent spinal influences, specific mechanisms usually cannot be isolated from amplitude changes alone. Conditioning paradigms, recruitment-curve analysis, and task-dependent modulation are therefore required to make mechanism-oriented inferences.

### 2.3. Control of Afferent Constancy and Stimulus Fidelity

To minimize confounding influences, contemporary protocols often evoke the control H-reflex on the ascending limb of the recruitment curve (Figure 2), typically at ~20–50% of H_max_, while maintaining a small, stable M-wave (often on the order of ~5–10% of M_max_). Stability of the M-wave serves as an indirect operational proxy for afferent constancy, indicating that a relatively consistent population of motor axons—and by inference, Ia afferents—is activated across trials [39,40]. Deviations in M-wave amplitude can compromise interpretability by altering the composition of the afferent volley rather than necessarily reflecting genuine modulation of spinal circuitry.

Although large H-reflexes can occasionally be elicited in the absence of a detectable M-wave, continuous M-wave monitoring remains recommended in most protocols. This practice helps to ensure that observed changes in reflex amplitude are more likely to reflect central modulatory mechanisms—such as presynaptic inhibition, homosynaptic depression, or recurrent inhibition—rather than changes in stimulus delivery or peripheral excitability. However, M-wave amplitude does not always guarantee identical recruitment of motor axons across trials, as it can be influenced by factors such as electrode placement, tissue conductivity, and changes in axonal excitability.

### 2.4. Re-Evaluating Motoneuron Excitability Metrics

The longstanding use of the H_max_/M_max_ ratio as an index of motoneuron pool excitability should be interpreted with caution. H_max_ is shaped by a confluence of pre- and postsynaptic mechanisms, including presynaptic inhibition, activity-dependent synaptic depression, recurrent inhibition, and antidromic collision in motor axons [1,9]. The H_max_/M_max_ ratio should therefore be interpreted as a composite index of reflex pathway excitability, not as a selective measure of α-motoneuron excitability.

A more informative approach is to consider distinctions between postsynaptic motoneuron readiness and presynaptic afferent efficacy, parameters that are difficult to isolate and are typically inferred through dynamic paradigms such as paired-pulse stimulation, frequency-dependent depression, or task-dependent modulation.

### 2.5. State Dependence and Task Specificity

Recording the H-reflex during low-level voluntary contraction (typically 5–10% of maximal voluntary contraction) can help stabilize motoneuron excitability and may improve within-subject reliability [6]. However, this approach also introduces descending drive and contraction-related sensory feedback, and modulates spinal inhibitory circuits, including presynaptic inhibition, recurrent inhibition, and Ib pathways [41,42].

Conversely, recordings obtained at rest minimize voluntary descending input but provide limited control over the baseline excitability of motoneurons and interneuronal networks, which may vary across individuals and recording sessions. Thus, neither condition can be considered physiologically neutral, and each entails specific advantages and limitations depending on the experimental or clinical question.

During voluntary movement, additional complexity arises from the recruitment and increased excitability of low-threshold motoneurons, which can influence reflex amplitude without necessarily reflecting proportional changes in force output [10]. Reflex gain varies across tasks, between agonist and antagonist muscles, and across contraction modes [43]. Transitions between rest and movement are associated with shifts in the Ia input–motoneuron output relationship, reflecting rapid reconfiguration of spinal circuitry [28,34,44]. Meaningful interpretation therefore requires explicit consideration of the motor, postural, and physiological context in which the reflex is elicited. These state-dependent influences, including rest versus voluntary contraction, posture, locomotion, task demands, attention, and training-related plasticity, represent an important source of variability in H-reflex measurements, with direct implications for experimental design, standardization, and interpretation, as discussed in the following section.

### 2.6. Determinants of H-Reflex Variability, Reliability, and Methodological Considerations

The amplitude and temporal characteristics of the H-reflex are highly sensitive to a range of experimental and physiological factors. While the preceding section addressed the physiological basis of state-dependent modulation, the present section focuses on how these and other factors influence the measurement, comparability, and interpretation of H-reflex responses in experimental and clinical settings. Accordingly, the methodological interpretation of H-reflex amplitude depends on controlling the main sources of variability, including stimulation intensity, recruitment-curve position, background EMG, posture, task conditions, and recent activation history [5,9,22,23,26,45,46,47].

A key determinant of H-reflex amplitude is the stimulation intensity relative to the recruitment curve. Because the H-reflex and M-wave have distinct recruitment properties, the position at which the reflex is sampled—whether on the ascending limb, near H_max_, or on the descending limb—affects both its magnitude and its responsiveness to modulation (Figure 2) [46]. The recruitment-curve approach, in which both H-reflex and M-wave responses are systematically characterized across stimulus intensities, has been widely recommended to identify stable operating points and reduce variability [23,48]. Experimental and reliability studies indicate that sampling on the ascending limb minimizes ceiling effects and improves sensitivity to physiological changes, whereas measurements near H_max_ are more variable. Small variations in stimulus intensity can alter the balance between afferent and motor axon recruitment, and stimulus drift across sessions is a major contributor to reduced test–retest reliability, even when M-wave amplitude appears stable [9,26]. Although normalization strategies such as the H_max_/M_max_ ratio are commonly used to reduce peripheral influences, they do not fully account for differences in recruitment dynamics and should be interpreted with caution [26,30]. Combining normalization approaches with recruitment-curve-derived parameters (e.g., slope or area) may improve robustness compared with single-point measures.

Background electromyographic activity is another major source of variability. Even small differences in baseline muscle activation can influence reflex size; therefore, protocols should standardize EMG levels or restrict analysis to predefined activation ranges [5,23]. When tonic contraction is used to improve stability, its level should be explicitly controlled and reported, because the resulting reflex represents a contraction-dependent condition rather than a resting measure [22].

Posture, joint angle, and motor or task-related context further influence reflex amplitude and variability. Differences between prone, seated, and standing conditions have been consistently reported, and reliability metrics vary across these conditions [5,23]. These factors should therefore be standardized or explicitly incorporated into the experimental design and interpretation.

The temporal structure of stimulation introduces additional variability through history-dependent effects such as homosynaptic (post-activation) depression and rate-dependent depression. Because these phenomena depend on prior activation and evolve over seconds, interstimulus interval and stimulation frequency must be carefully controlled and reported [7,11,49]. Low-frequency stimulation is typically used to assess baseline excitability, whereas higher-frequency paradigms are used to probe inhibitory mechanisms. Recent methodological work emphasizes the need for standardized protocols for rate-dependent depression, as heterogeneity in stimulation parameters limits comparability. Simplified paradigms using short stimulus trains at predefined frequencies have shown improved feasibility and reproducibility while preserving sensitivity to spinal inhibitory processes [17,50,51].

Muscle selection and anatomical factors further limit comparability across studies. Muscles differ in spindle density, functional role, and descending control, leading to differences in H-reflex amplitude, stability, and modulation. The soleus typically exhibits a more robust and stable H-reflex than upper limb muscles such as the flexor carpi radialis, and findings cannot be directly generalized across muscles [5,29]. Electrode placement and stimulation site are also critical determinants of signal quality and reproducibility. Even small shifts in electrode position can alter recorded amplitudes, and standardized placement improves between-session reliability [5,26]. Although M-wave amplitude is commonly used as an indicator of stimulation consistency, it does not guarantee identical afferent recruitment or engagement of spinal circuits [22,26].

Trial-to-trial variability necessitates averaging of repeated responses to obtain reliable estimates. Classical recommendations suggest averaging multiple stimuli per condition, and increasing the number of trials improves within-session stability and between-session reliability [5,22]. More recent evidence indicates that reliable estimates can often be achieved with a moderate number of repetitions when other sources of variability are well controlled, and that recruitment-curve-based sampling strategies may further enhance efficiency.

Additional variability arises from inter-individual differences and the absence of universally accepted normative values. H-reflex measures vary with demographic and physiological factors, including age, sex, and physical conditioning. Aging is associated with prolonged latency and variable changes in amplitude and activity-dependent depression, reflecting combined peripheral and central mechanisms [52,53,54]. Accordingly, within-subject comparisons under standardized conditions are generally more informative than between-subject comparisons using fixed thresholds [5,29].

Test–retest reliability studies provide important insight into the robustness of H-reflex measurements. Under well-controlled conditions, moderate to high reliability has been reported for normalized H-reflex amplitudes and recruitment-curve parameters, with intraclass correlation coefficients often exceeding 0.7 [22,26,47,55]. Reliability is typically higher within sessions than between sessions and normalized and recruitment-based measures outperform absolute amplitudes, whereas latency measures tend to be more stable. These findings support the use of standardized protocols, normalization strategies, and within-subject designs to improve interpretability.

Despite these methodological approaches, H-reflex measurements remain inherently context-dependent, and careful control and reporting of experimental conditions are essential for meaningful comparisons across trials, sessions, and subjects (Table 1).

## 3. The H-Reflex as a Non-Invasive Probe of Spinal Circuitry and Segmental Neurophysiology

Changes in H-reflex amplitude following a conditioning stimulus have long been used to infer, indirectly, the functional state of specific spinal pathways. Because the test reflex depends on both α-motoneuron responsiveness and presynaptic modulation of Ia afferent terminals, conditioning paradigms must be interpreted as changes in the net reflex pathway rather than as isolated measures of a single synaptic process.

Formally, the amplitude of the H-reflex (H) can be conceptualized as being proportional to:

(1) $$H\propto \sum_{i=1}^{N}P_{i}\cdot Q_{i}\cdot S_{i},$$

where $P_{i}$ denotes the probability of neurotransmitter release at the Ia terminal contacting motoneuron i, $Q_{i}$ represents postsynaptic responsiveness (membrane potential relative to threshold and synaptic conductance state), and $S_{i}$ reflects the probability that the motoneuron lies within the subliminal fringe. This formulation is intended as a conceptual framework rather than a quantitatively exact model, as its components cannot be independently measured and it assumes linear summation of motoneuron contributions, whereas synaptic integration and motor unit recruitment exhibit nonlinear dynamics. Conditioning paradigms can be interpreted as influencing one or more of these factors, thereby providing a structured way to consider the multiple determinants of H-reflex amplitude.

### 3.1. Monosynaptic Ia Excitation and Homosynaptic (Post-Activation) Depression

The amplitude of the H-reflex depends in part on the recent activation history of the Ia afferents mediating the test volley, even when stimulus intensity, background EMG, and motoneuron recruitment gain are held constant. This phenomenon, termed homosynaptic depression or post-activation depression, occurs at the synapse between Ia afferents and α-motoneurons and represents a well-established form of short-term synaptic plasticity within spinal reflex pathways.

Classic intracellular recordings in the cat demonstrated that repetitive low-frequency activation of Ia afferents (for example, ~3 impulses·s^−1^) produces a progressive reduction in the amplitude of monosynaptic EPSPs, attributable to a decrease in neurotransmitter release from presynaptic terminals [59]. Subsequent work in humans provided evidence consistent with an analogous mechanism in the soleus H-reflex: post-activation depression occurs without measurable changes in motoneuron membrane potential or firing threshold, supporting a predominantly presynaptic contribution [7,8].

Quantitatively, homosynaptic depression (HSD) can be operationally expressed as:

(2) $${HSD}_{n}=1-\frac{H_{n}}{H_{1}},$$

where $H_{1}$ is the amplitude of the first reflex and $H_{n}$ the amplitude of the n-th reflex. This index provides a descriptive measure of relative depression, not a direct quantification of synaptic mechanisms. Recovery from depression follows a slow exponential time course:

(3) $$H(t)=H_{1}\left(1-A\cdot e^{-t/\tau_{rec}}\right),$$

where A denotes depression magnitude and $\tau_{rec}$ the recovery time constant, typically on the order of several seconds. This formulation is an empirical approximation, as recovery dynamics may vary across conditions and individuals. Accordingly, when consecutive H-reflexes are elicited at short inter-stimulus intervals (1–2 s), depression is pronounced, whereas near-complete recovery typically requires intervals approaching 8–10 s [3,8].

Homosynaptic depression is strongly state-dependent. It is reduced during voluntary contraction of the homonymous muscle and has been reported to be markedly attenuated or absent during standing when soleus activity reaches approximately 15–20% of maximal voluntary contraction [60]. In addition to electrical activation, post-activation depression may arise from passive muscle stretch, voluntary contraction, or Ia afferent discharge elicited during antagonist muscle activity [7]. Recent evidence further indicates that passive muscle lengthening decreases the effectiveness of Ia afferents in discharging α-motoneurons across different muscle lengths, partly through increased homosynaptic post-activation depression, with longer muscle lengths also modulating primary afferent depolarization and heteronymous Ia facilitation [61]. To avoid contamination by movement-induced depression, test and conditioning stimuli are typically delivered either at movement onset or following sufficiently long quiescent periods.

Clinically, reduced homosynaptic depression has been associated with muscle stiffness, spasticity, and hyperreflexia following spinal cord injury, stroke, and other supraspinal lesions, as well as in animal models of spinal contusion [3,4,62]. Despite its clear relevance to pathological motor states, the precise functional role of homosynaptic depression in normal human motor control remains incompletely understood.

### 3.2. Presynaptic Inhibition of Ia Afferents

Sensory afferent input from skin, muscles, tendons, and joints continuously converges onto spinal circuits. Effective motor control requires that this inflow be selectively gated to prevent destabilization of motor output. A key site for such gating is the presynaptic terminal of primary afferents.

Presynaptic inhibition was first characterized by Frank and Fuortes [63], who demonstrated depression of EPSP amplitude in the absence of changes in postsynaptic membrane potential or excitability. This form of inhibition is mediated by predominantly GABAergic axo-axonal synapses that induce primary afferent depolarization, thereby modulating calcium entry and neurotransmitter release from Ia terminals [2]. At the receptor level, this process should not be reduced to a generic GABAergic effect. Classical primary afferent depolarization is mainly associated with GABA_A_ receptor-mediated mechanisms, whereas GABA_B_ receptors may also contribute to presynaptic modulation through slower metabotropic effects on calcium entry and transmitter release. Interneurons mediating presynaptic inhibition are activated by group I afferents, inhibited by flexor reflex afferents, and subject to strong descending control [64]. Importantly, routine H-reflex recordings in humans cannot directly separate GABA_A_- from GABA_B_-mediated contributions and should therefore be interpreted as indirect measures of presynaptic control.

Presynaptic inhibition is a major contributor to the modulation of monosynaptic reflexes across a wide range of motor or postural states. In humans, changes in presynaptic inhibition have been associated with modulation of the soleus H-reflex during passive limb movement, joint rotation, posture, and locomotion [65,66,67]. Importantly, presynaptic inhibition can alter reflex amplitude independently of α-motoneuron excitability, which helps explain why identical background EMG levels may be associated with markedly different H-reflex amplitudes across tasks [34].

At the onset of voluntary contraction, presynaptic inhibition acting on Ia afferents projecting to the contracting muscle is often reduced, likely through descending cortical mechanisms [68,69]. This reduction is selective: Ia afferents projecting to antagonist motoneuron pools may remain inhibited or even experience enhanced inhibition, facilitating appropriate agonist–antagonist coordination [70].

Presynaptic inhibition cannot be measured directly in humans and must therefore be inferred from indirect, well-validated paradigms. These include comparisons between changes in background EMG and H-reflex amplitude [71], vibration-induced reflex depression, and conditioning–test paradigms involving heteronymous afferent volleys.

Electrical stimulation of the common peroneal or radial nerve evokes multiple phases of H-reflex depression in antagonist muscles. Depression appearing at conditioning–test intervals of approximately 6–30 ms, termed D1 inhibition, is commonly interpreted as being largely mediated by presynaptic inhibition of Ia afferents [10,72]. Longer-lasting depression (up to ~200 ms) likely reflects contributions from additional polysynaptic inhibitory circuits.

A conceptually distinct approach involves heteronymous Ia facilitation. Conditioning stimulation of the femoral nerve evokes monosynaptic excitation of soleus motoneurons via heteronymous Ia projections. Changes in facilitation within the first ~0.5 ms have been used as an indirect index of ongoing presynaptic inhibition:

(4) $$\Delta H_{hetero}\propto -{PI}_{Ia},$$

where $\Delta H_{hetero}$ denotes the change in H-reflex amplitude produced by heteronymous Ia facilitation, and ${PI}_{Ia}$ denotes presynaptic inhibition acting on Ia afferent terminals. The negative proportionality indicates that larger heteronymous facilitation is interpreted as reflecting lower ongoing Ia presynaptic inhibition [42]. This relationship should be interpreted qualitatively rather than as a strict quantitative law, as both facilitation and inhibition are influenced by multiple converging mechanisms. Crucially, heteronymous facilitation and D1 inhibition probe different aspects of presynaptic control: the former reflects tonic or ongoing inhibitory tone, whereas the latter reflects the presynaptic inhibitory network’s responsiveness to conditioning input.

### 3.3. Reciprocal Ia Inhibition

Reciprocal Ia inhibition is a key mechanism contributing to coordinated motor control. Ia afferents from a contracting muscle excite inhibitory interneurons that suppress motoneurons innervating the antagonist muscle. This pathway, involving Ia inhibitory interneurons, receives convergent input from corticospinal, rubrospinal, vestibulospinal, and segmental afferent systems [64,73].

In humans, reciprocal inhibition can precede antagonist muscle activation by up to 50 ms, suggesting a contribution of supraspinal mechanisms and anticipatory control [74]. Quantitatively, reciprocal inhibition is commonly estimated as:

(5) $$RI=1-\frac{H_{cond}}{H_{test}},$$

where $H_{test}$ is the unconditioned H-reflex amplitude and $H_{cond}$ the conditioned response. This index provides a relative measure of inhibition and should be interpreted descriptively, as it does not isolate specific underlying mechanisms.

Interpretation of reciprocal inhibition requires careful control of conditioning stimulus intensity, reflex size, and the motor or postural state in which the reflex is elicited. At higher conditioning intensities, the stimulus may recruit not only antagonist Ia afferents but also Ib or other large-diameter sensory afferents, which can influence the test H-reflex through non-reciprocal inhibitory or polysynaptic pathways. Under these conditions, the resulting H-reflex modulation should not be interpreted as a selective measure of reciprocal Ia inhibition.

### 3.4. Non-Reciprocal (Ib) Inhibition

Golgi tendon organs provide force-related feedback via Ib afferents, which engage di- and trisynaptic pathways that can inhibit synergistic motoneurons and facilitate or inhibit antagonists. Initially considered protective mechanisms, Ib pathways are now recognized as important contributors to the regulation of muscle stiffness and load-dependent control [75].

Ib inhibition is task-dependent. During locomotion, group I afferents from ankle extensors may contribute to reinforcing extensor activity and stabilizing the stance phase, whereas during static conditions, Ib inhibition is more often associated with limiting excessive force production [76,77]. In humans, short-latency Ib inhibition is typically assessed by conditioning the soleus H-reflex with stimulation of the medial gastrocnemius nerve at low stimulus intensities and conditioning–test intervals of approximately 6 ms, although separation from Ia-mediated effects and other group I contributions may not be complete.

### 3.5. Recurrent Inhibition

Recurrent inhibition mediated by Renshaw cells provides a form of local feedback regulation of motoneuron discharge. Renshaw cells receive input from motor axon collaterals and project to α- and γ-motoneurons, Ia inhibitory interneurons, and other Renshaw cells, integrating segmental and descending inputs [78,79].

Recurrent inhibition has been proposed to influence motoneuron gain, discharge synchronization, and the selection of synergies. Its net effect on motor output can be conceptually approximated as:

(6) $$G_{eff}=\frac{G_{0}}{1+\beta }\;,$$

where $G_{0}$ is intrinsic motoneuron gain, and β represents recurrent inhibitory strength. This expression is a simplified conceptual representation and does not correspond to a directly measurable or isolated physiological parameter. Task-dependent modulation of β may contribute to stabilizing output during weak contractions and facilitating force production during stronger contractions.

Taken together, conditioning paradigms of the H-reflex provide indirect access to multiple inhibitory processes within spinal circuitry, but their mechanistic interpretation remains inferential and context-dependent. Homosynaptic depression, presynaptic inhibition, reciprocal inhibition, Ib-mediated load feedback, and recurrent inhibition are not isolated phenomena but interacting components of a flexible spinal control system. Accordingly, interpretation of H-reflex modulation requires careful consideration of recruitment conditions, afferent input, and the motor, postural, and task-related context in which the reflex is elicited, as discussed in previous sections.

## 4. Classical Clinical Applications of the H-Reflex

The Hoffmann reflex has long been used in clinical neurophysiology as a tool to assess the functional integrity of peripheral and spinal pathways (Table 2). Its principal value lies in providing indirect access to proximal segments of the reflex arc, which are not readily evaluated using conventional nerve conduction studies. In particular, H-reflex latency has been widely employed in the assessment of S1 radiculopathy through soleus recordings, as well as in the evaluation of proximal conduction in upper limb pathways such as the flexor carpi radialis (FCR) [9,29]. Because the H-reflex traverses the entire monosynaptic reflex arc—including Ia afferents, spinal synapses, and α-motoneurons—it offers a clinically useful measure of conduction along segments that are otherwise difficult to assess electrophysiologically. However, latency measures are influenced by physiological factors such as height, limb length, and age, which must be considered when interpreting results [29,45].

Beyond conduction studies, the H-reflex has also been used as an index of reflex excitability, particularly in conditions characterized by altered descending control, such as spasticity and upper motor neuron syndromes. Here, upper motor neuron syndromes refer to disorders caused by lesions or dysfunction of descending motor pathways, particularly corticospinal and related supraspinal systems, and clinically characterized by combinations of spasticity, hyperreflexia, clonus, abnormal reflex spread, weakness, and impaired voluntary motor control. In this context, the ratio of maximal H-reflex amplitude to maximal M-wave amplitude (H_max_/M_max_) has historically been interpreted as reflecting the excitability of the motoneuron pool. Increased ratios have been associated with hyperreflexia and reduced inhibitory control, whereas reduced ratios have been linked to diminished reflex responsiveness [5,30]. Nevertheless, as discussed in previous sections, it is now well recognized that this metric lacks specificity, as H-reflex amplitude is influenced by multiple factors beyond motoneuron excitability, including presynaptic inhibition and activity-dependent modulation of Ia afferent transmission [9,23]. Accordingly, while the H_max_/M_max_ ratio remains widely reported, its interpretation requires caution.

More broadly, the H-reflex has served as a tool for assessing segmental spinal function, providing insight into the integrity of Ia afferent pathways and the responsiveness of α-motoneuron pools. Abnormalities in H-reflex latency, amplitude, or recruitment have been described in a range of neurological conditions, including peripheral neuropathies, radiculopathies, and spinal cord lesions, and have been used to aid in the differential diagnosis of central versus peripheral disorders [5,29]. However, consistent with earlier discussion, the H-reflex does not represent a purely monosynaptic response and is modulated by both spinal circuitry and experimental conditions, limiting its ability to provide unequivocal mechanistic interpretations [9,23].

These classical applications established the H-reflex as a versatile clinical tool for probing conduction, excitability, and segmental spinal function. At the same time, the recognized limitations have motivated the development of more refined paradigms aimed at isolating distinct components of spinal circuit function.

## 5. Emerging Translational Applications: H-Reflex—Based Assessment of Spinal Inhibitory Function

Whereas classical clinical applications of the H-reflex have primarily focused on latency measures and gross indices of reflex excitability, recent work has increasingly leveraged the H-reflex as a probe of spinal inhibitory control. In this context, paradigms assessing homosynaptic (post-activation) depression and related phenomena, such as rate-dependent depression, have emerged as useful approaches to evaluate the functional state of spinal inhibitory circuits in humans.

The Hoffmann reflex is one of the few non-invasive electrophysiological tools that provides indirect functional information about human spinal reflex circuitry. Conceptually, it probes the transmission of Ia afferent input onto α-motoneurons while simultaneously capturing the influence of multiple inhibitory and facilitatory mechanisms, including presynaptic inhibition, homosynaptic depression, reciprocal Ia inhibition, Ib inhibition, and recurrent inhibition mediated by Renshaw cells [5,7].

Unlike peripheral nerve conduction studies, which primarily assess axonal conduction properties, or transcranial magnetic stimulation, which reflects corticospinal and intracortical excitability, the H-reflex provides indirect access to spinal sensorimotor integration. This property has led to its use as a potential translational bridge between basic spinal neurophysiology and clinical neurology, although its interpretation requires careful consideration of the multiple mechanisms contributing to the recorded response [6,80].

As discussed in previous sections, H-reflex amplitude does not represent a unitary measure of motoneuron excitability. Rather, it reflects the interaction between motoneuron membrane properties, synaptic efficacy at Ia afferent–α-motoneuron synapses, and the dynamic modulation exerted by spinal interneuronal networks [1,9]. Recognizing this complexity is essential when interpreting H-reflex-based measures in clinical and translational contexts.

### 5.1. Chronic Pain Syndromes and Spinal Disinhibition

In painful diabetic peripheral neuropathy, fibromyalgia, and other chronic pain states, several studies report attenuation of rate-dependent depression (RDD) of the H-reflex, consistent with impaired homosynaptic depression at Ia afferent terminals [15,16]. RDD has been interpreted as reflecting alterations in spinal inhibitory control, in which repetitive afferent input fails to dampen synaptic transmission, potentially contributing to increased sensory gain.

This mechanism provides a plausible neurophysiological explanation for pain syndromes that are poorly correlated with peripheral nerve degeneration and often refractory to conventional analgesics. Importantly, pharmacological studies have reported that agents with proven efficacy in neuropathic pain, such as pregabalin, are associated with partial normalization of RDD, whereas ineffective compounds do not [51,81,82]. These findings suggest that H-reflex-based measures may provide mechanism-informed markers of treatment response, rather than serving solely as nonspecific indicators of disease severity.

### 5.2. Motor Disorders, Spasticity, and Recovery After Injury

After stroke or spinal cord injury, reductions in homosynaptic depression and abnormalities in reciprocal and recurrent inhibition have been reported to be associated with spasticity, impaired motor selectivity, and abnormal muscle synergies [3,4,83,84]. These findings are not fully explained by the traditional view that spasticity arises solely from increased motoneuron excitability and instead are consistent with a contribution of altered inhibitory spinal networks to the regulation of reflex gain.

Longitudinal studies suggest that recovery of homosynaptic depression may parallel improvements in voluntary motor function and gait performance, although this relationship is not uniform across individuals or conditions [60,62,85,86]. From a translational perspective, H-reflex metrics may provide physiologically relevant endpoints for rehabilitation trials, neuromodulation strategies, and pharmacological interventions targeting spinal plasticity, provided that their limitations and context dependence are carefully considered.

### 5.3. Parkinson’s Disease

In Parkinson’s disease, alterations in H-reflex modulation during voluntary contraction and postural control tasks have been reported and appear to depend on both motor state and dopaminergic tone, reflecting complex interactions between descending control and spinal inhibitory circuits [18,87,88]. Specifically, postactivation depression is reduced at rest in untreated patients and can be restored by dopaminergic medication or deep brain stimulation, indicating a modulatory influence of basal ganglia output on spinal inhibitory mechanisms [18].

During functional tasks such as gait initiation, task-dependent modulation of the H-reflex is also altered, with the degree of soleus H-reflex inhibition correlating with disease severity and measures of motor impairment [87]. These findings suggest that abnormalities in descending motor commands may contribute to impaired regulation of spinal inhibitory circuits during movement.

These alterations have been associated with rigidity, impaired postural reflexes, and pain—symptoms that are not fully accounted for by basal ganglia dysfunction alone. However, the extent to which spinal mechanisms independently contribute to these clinical features remains incompletely established.

The H-reflex thus provides a framework to examine the potential contribution of spinal circuitry to motor control abnormalities in Parkinson’s disease, particularly in relation to state-dependent modulation and treatment effects. This perspective is consistent with models that emphasize distributed network dysfunction across cortical, subcortical, and spinal levels [1].

### 5.4. Quantitative Definitions and Electrophysiological Parameters

To facilitate clinical translation, H-reflex-derived measures require clear and standardized operational definitions:

H-reflex amplitude (H): Peak-to-peak electromyographic response elicited by submaximal stimulation of a mixed peripheral nerve, commonly normalized to maximal M-wave amplitude (M_max_) to partially control for peripheral excitability and recording conditions [9,22,23].

**Figure 3:**
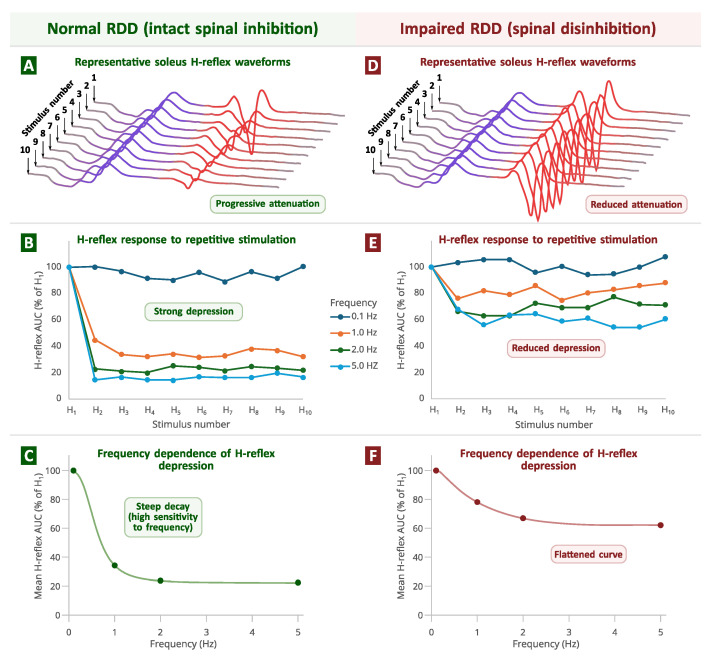
Rate-dependent depression (RDD) of the H-reflex under normal and impaired spinal inhibitory conditions. Panels (**A**–**C**) illustrate normal RDD, reflecting intact spinal inhibitory control. (**A**) Representative soleus H-reflex waveforms evoked by repetitive stimulation show progressive attenuation across stimuli, consistent with post-activation (homosynaptic) depression. (**B**) Quantification of H-reflex amplitude (AUC normalized to H_1_) demonstrates strong depression at higher stimulation frequencies. (**C**) The frequency–response relationship reveals a steep decay, indicating high sensitivity of the reflex to stimulation rate. Panels (**D**–**F**) depict impaired RDD, consistent with spinal disinhibition. (**D**) Representative waveforms show reduced attenuation across stimuli. (**E**) H-reflex amplitude remains elevated during repetitive stimulation, reflecting diminished depression. (**F**) The frequency–response curve is flattened, indicating reduced sensitivity to stimulation frequency. AUC, area under the curve; H_1_, first H-reflex response evoked in a train of stimuli.

Rate-dependent depression (RDD):

(7) $$\mathrm{RDD}\left(\%\right)=\left(1-\frac{H_{f}}{H_{1}}\right)\times 100,$$

where $H_{1}$ is the first H-reflex amplitude and $H_{f}$ is the mean amplitude of subsequent responses at a given stimulation frequency. This measure reflects the progressive reduction in reflex amplitude with repeated activation, largely attributed to post-activation (homosynaptic) depression, although additional mechanisms may contribute depending on the protocol (Figure 3) [7,15].

Homosynaptic depression index: Ratio of conditioned to unconditioned H-reflex amplitude at inter-stimulus intervals of 1–10 s, interpreted as reflecting activity-dependent changes at Ia afferent terminals, often attributed to reduced transmitter release, although not exclusively presynaptic in origin [8,49].

Presynaptic inhibition (D1 inhibition): Reduction in test H-reflex amplitude at conditioning–test intervals of approximately 6–30 ms following heteronymous nerve stimulation, mainly through GABA_A_ receptor-mediated primary afferent depolarization at Ia afferent terminals, although GABA_B_-mediated presynaptic modulation and task-dependent or segmental factors may also contribute [2,72].

When derived under controlled conditions, these parameters can provide relatively reproducible indices of spinal inhibitory function, although their interpretation remains context-dependent and influenced by multiple physiological and methodological factors [5,22,23].

## 6. Translational Gaps and Methodological Challenges

Despite its well-established physiological basis, the clinical translation of H-reflex-derived measures remains limited by insufficient standardization, incomplete reliability data, and limited inter-laboratory comparability [5]. These limitations are particularly relevant for dynamic paradigms, such as rate-dependent depression, conditioning protocols, and recruitment-curve metrics, where small differences in acquisition or analysis can alter physiological interpretation. Reproducible implementation requires explicit definition of the neurophysiological setting in which the reflex is obtained. At minimum, studies should report the target muscle and stimulated nerve, stimulation site, electrode montage, body position, joint angle, background electromyographic activity or voluntary contraction level, stimulus pulse duration and waveform, position on the recruitment curve, M-wave criteria for stimulus constancy, interstimulus interval or stimulation frequency, number of trials, averaging procedure, and normalization strategy. In clinical studies, medication state, neuromodulation status, fatigue, pain, spasticity severity, disease stage, and time since neurological injury should also be specified when relevant.

A major consequence of protocol heterogeneity is that H-reflex outcomes may differ across laboratories for methodological rather than physiological reasons. Although recent studies have proposed reduced, time-efficient, and optimized protocols to improve feasibility in clinical and translational settings, these approaches require further validation in independent cohorts and across different muscles, populations, and disease contexts [50,82,89,90,91,92]. In particular, test–retest reliability, within-subject variability across sessions, and between-laboratory reproducibility remain incompletely characterized for several H-reflex-derived metrics. Establishing such reliability is essential before these measures can be used confidently in longitudinal monitoring, interventional trials, or individual patient stratification.

Normative reference frameworks also remain insufficient. Unlike peripheral nerve conduction studies, which are supported by well-established reference ranges, many H-reflex-derived measures lack large age-, sex-, height-, limb-, and muscle-specific datasets. This limitation is important because H-reflex presence, amplitude, latency, and modulation vary across muscles, recording conditions, and populations. The soleus and triceps surae are common and relatively well-characterized targets, but H-reflexes can also be recorded from other upper- and lower-limb muscles when appropriate protocols and reference values are available. Without robust normative datasets, H-reflex abnormalities often remain descriptive or group-level observations rather than quantitative markers that can guide individual clinical interpretation.

Another operational challenge is context dependence. H-reflex amplitude and modulation vary with posture, joint configuration, background muscle activation, task demands, locomotor phase, attention or arousal, and recent activation history. This context dependence is physiologically meaningful, because it reflects adaptive regulation of spinal sensorimotor circuits in awake, behaviorally relevant conditions. However, it also complicates clinical inference. To distinguish pathological disinhibition from appropriate context-dependent modulation, protocols should avoid pooling recordings obtained under different motor or postural conditions, and should explicitly define whether measures were obtained at rest, during voluntary contraction, standing, locomotion, or another task condition [6,34]. Identical H-reflex amplitudes may have different physiological meanings depending on these conditions.

Finally, the mechanistic specificity of H-reflex-derived measures remains limited. Conventional H-reflex amplitude reflects a net reflex output shaped by afferent recruitment, Ia afferent–α-motoneuron synaptic efficacy, presynaptic and postsynaptic modulation, interneuronal networks, descending drive, and α-motoneuron excitability. Therefore, isolated H-reflex amplitude should not be interpreted as a disease-specific biomarker or as a direct measure of a single inhibitory mechanism. More standardized dynamic and conditioning paradigms may provide more mechanistically informative candidate markers, but their interpretation still requires caution. Integrative approaches combining H-reflex paradigms with clinical phenotyping, neuroimaging, pharmacological testing, molecular biomarkers, or other neurophysiological measures may help link circuit-level abnormalities with underlying synaptic and molecular mechanisms [1,82]. However, the feasibility and added value of such multimodal approaches in routine clinical settings remain to be established.

Viewed in this operational framework, the H-reflex remains a valuable experimental and translational tool for assessing spinal reflex pathway function. Its clinical utility will depend less on treating H-reflex amplitude as a standalone measure and more on implementing standardized protocols, reporting the relevant neurophysiological context, developing robust normative datasets, and validating reliability across sessions, laboratories, muscles, and clinical populations. Addressing these requirements will be essential for clarifying the role of H-reflex-derived measures in clinical neurology, rehabilitation, and mechanism-based approaches to neurological disease.

## 7. Discussion

This review positions the H-reflex as a mechanism-sensitive probe of human spinal circuit function rather than solely a classical experimental measure. When interpreted within a systems-level framework that recognizes the spinal cord as a dynamic and adaptable component of motor control, H-reflex-based paradigms provide access to the regulation of sensorimotor gain, inhibitory control, and aspects of activity-dependent modulation in the intact human nervous system [1,9]. The evidence reviewed here supports moving beyond simplified interpretations of reflex amplitude toward a more integrative, circuit-level perspective.

A recurring observation across physiological and clinical studies is that alterations in spinal inhibitory processes are present in conditions traditionally attributed primarily to supraspinal dysfunction. Changes in homosynaptic depression, presynaptic inhibition, and interneuronal modulation have been described in chronic neuropathic pain, spasticity after central injury, and movement disorders, including Parkinson’s disease [3,4,93]. These findings suggest that spinal mechanisms contribute to clinical manifestations, although their relative role likely varies across conditions. In this context, the H-reflex provides a practical experimental window into human spinal physiology that complements supraspinal measures such as transcranial magnetic stimulation or functional neuroimaging [6].

From a translational perspective, one of the more consistent findings is the sensitivity of H-reflex-derived measures to interventions that modulate neural excitability. RDD has been proposed as an index of spinal inhibitory function, particularly in studies of painful diabetic neuropathy and related conditions [15]. Some pharmacological studies report partial normalization of RDD with agents such as gabapentinoids, whereas other compounds show limited effects [17,81]. While these observations support the potential utility of H-reflex-based measures as markers of treatment-related physiological changes, their specificity and predictive value at the individual level remain to be fully established.

Despite these strengths, several conceptual and methodological challenges continue to constrain broader adoption of H-reflex-based measures.

### 7.1. Conceptual Challenges: Abandoning Motoneuron-Centric Interpretations

The most persistent conceptual limitation is the continued interpretation of the H-reflex as a proxy for α-motoneuron excitability. As demonstrated repeatedly, reflex amplitude reflects a composite of presynaptic neurotransmitter release, interneuronal inhibition, and the dynamic size of the subliminal fringe rather than a unitary postsynaptic parameter [1,9]. Ratios such as H_max_/M_max_, when interpreted without mechanistic context, may obscure the inhibitory processes that render the H-reflex informative. Progress in the field therefore requires explicit recognition that H-reflex measures index the net state of spinal circuitry more than motoneuron output per se.

Closely related is the issue of context dependence. Reflex amplitude and modulation vary systematically with posture, background muscle activation, task demands, arousal or attention, locomotor phase, and recent sensory history, reflecting adaptive reconfiguration of spinal circuitry rather than experimental noise [34,60]. Although often viewed as a methodological nuisance, this property can also be considered a strength of the H-reflex, because it captures spinal control in awake, behaviorally relevant conditions. The operant conditioning studies of Wolpaw and colleagues provide an important example of this principle, showing that the H-reflex can be bidirectionally modified through training and that such changes involve plasticity within spinal reflex pathways and their descending regulation [94,95]. The challenge lies in distinguishing pathological loss of inhibitory control from appropriate task-dependent modulation, a distinction that requires careful experimental design and transparent reporting rather than oversimplification.

### 7.2. Methodological Challenges: Standardization Without Loss of Physiological Meaning

A major obstacle to clinical translation remains methodological heterogeneity. Studies differ widely in stimulation frequency, selection of reflex size, contraction level, and normalization strategy, limiting comparability across laboratories and populations [5]. Unlike peripheral nerve conduction studies, which benefit from decades of standardization and normative datasets, H-reflex methodologies still lack broadly adopted consensus frameworks suitable for multicenter application.

Crucially, standardization must not come at the expense of physiological interpretability. Overly rigid protocols risk reducing sensitivity to context-dependent modulation, whereas excessive flexibility undermines reproducibility. A pragmatic path forward may involve tiered approaches that distinguish core clinical metrics—such as RDD assessed under defined conditions—from more detailed paradigms designed to probe specific inhibitory mechanisms [5,7]. Recent efforts to develop reduced, time-efficient acquisition protocols are encouraging, but further validation across different populations and experimental conditions remains necessary.

A further limitation is the lack of large, age- and sex-stratified normative datasets. Without robust reference ranges, interpretation remains largely relative and group-based, restricting individual-level clinical inference. Establishing such datasets would represent an important step, analogous—although not directly equivalent—to the development of normative values for electroencephalography or nerve conduction studies.

### 7.3. Mechanistic Specificity and Cross-Scale Integration

Although H-reflex abnormalities provide indirect evidence of altered spinal inhibitory control, they do not uniquely identify the underlying molecular substrates. Presynaptic inhibition, homosynaptic depression, and recurrent inhibition involve partially overlapping but distinct neurotransmitter systems and interneuronal populations [2,64]. As a result, changes in H-reflex-derived measures cannot be straightforwardly attributed to a single synaptic mechanism. Addressing this limitation will likely require integrative approaches combining H-reflex paradigms with pharmacological manipulation, neuroimaging, and molecular biomarkers to better relate circuit-level observations to synaptic and cellular processes [1,17,81].

This perspective is broadly consistent with contemporary neuroscience approaches that aim to relate observations across multiple levels of organization. Within this context, the spinal cord can be viewed as an active component of sensorimotor processing whose dysfunction may contribute to both motor and non-motor features, although its relative role should be interpreted alongside supraspinal mechanisms.

### 7.4. Interpretative Limits and Areas of Ongoing Debate in H-Reflex Research

Despite its extensive use as a probe of spinal function, the H-reflex is subject to important interpretative limitations and inferential constraints. These arise from the indirect nature of the measure and the overlapping physiological mechanisms that contribute to its modulation. Recognizing these limitations is essential to appropriately position H-reflex-based findings within broader neurophysiological frameworks [5,9].

A central challenge in interpreting H-reflex modulation lies in the ambiguity of mechanistic attribution, particularly in distinguishing between presynaptic inhibition and homosynaptic (post-activation) depression. Although these mechanisms arise from distinct physiological processes, they can coexist and produce similar changes in reflex amplitude. In humans, standard H-reflex paradigms provide limited ability to dissociate these contributions; therefore, changes in amplitude cannot be unequivocally attributed to a single inhibitory pathway without additional experimental controls or complementary approaches [5,7].

More broadly, the H-reflex reflects the integrated output of multiple spinal and descending influences rather than a purely monosynaptic response. Contributions from reciprocal Ia inhibition, Ib inhibition, recurrent inhibition, and supraspinal inputs can all shape the final motor output [9,23]. This integrative nature complicates efforts to infer specific circuit mechanisms from changes in reflex amplitude.

These interpretative constraints are particularly relevant in the context of emerging translational applications. For example, the use of RDD as an index of altered spinal inhibitory function in conditions such as chronic pain and neuropathy has generated increasing interest. However, its specificity and mechanistic basis remain under investigation, and it is not yet fully resolved to what extent RDD reflects presynaptic inhibition, homosynaptic depression, or broader alterations in spinal network function [17,81]. Similarly, the relative contribution of spinal versus supraspinal mechanisms to H-reflex modulation continues to be discussed, particularly in paradigms involving voluntary contraction, posture, or neuromodulatory interventions, where descending influences may substantially shape the observed responses [9,23].

## 8. Conclusions

In summary, the evidence reviewed here supports a reconsideration of the H-reflex as a context-dependent probe of human spinal circuit function. Rather than reflecting a single physiological process, H-reflex-derived measures capture the integrated influence of multiple spinal and descending mechanisms, which can provide insight into the regulation of sensorimotor function when interpreted within an appropriate framework.

The factors that currently limit broader clinical application—including conceptual ambiguity, methodological variability, and limited mechanistic specificity—largely reflect the complexity of the underlying neurophysiology and the constraints of indirect measurement. Addressing these challenges will require continued efforts toward methodological harmonization, improved characterization of reliability and normative variability, and integration with complementary approaches capable of linking circuit-level observations to underlying biological mechanisms.

Within these boundaries, the H-reflex remains a useful experimental and translational tool. Its value is likely to lie not in providing definitive mechanistic attribution, but in contributing to multimodal assessments of spinal function across physiological and clinical contexts. Continued refinement of its methodological and interpretative framework may help clarify its role in both research and selected clinical applications.

## Figures and Tables

**Table 1:** Methodological factors influencing H-reflex.

Factor	Mechanistic Effect on H-Reflex	Impact on Measurement	Recommended Control/Standardization
Stimulation intensity [22,23]	Determines relative recruitment of Ia afferents and motor axons; defines position on recruitment curve	Major determinant of amplitude and variability; influences responsiveness to modulation	Normalize stimulus intensity relative to M_max_ or H_max_; ensure stable stimulation conditions across trials
Position on recruitment curve [26,39]	Reflects proportion of motoneuron pool activated and balance between Ia input and antidromic collision	Influences both amplitude and sensitivity to facilitation or inhibition	Sample consistently at a defined point on the curve; ascending limb is often used to reduce saturation effects
Electrode placement (stimulating and recording) [22,32]	Affects current distribution and motor unit detection	Source of inter-session and inter-subject variability	Standardize electrode location using anatomical landmarks; maintain consistent montage and impedance
Peripheral stimulation parameters (pulse duration, waveform) [22,30]	Influence fiber recruitment selectivity (Ia vs. motor axons)	Can alter recruitment characteristics and comparability across sessions	Use consistent pulse duration and waveform across conditions
M-wave monitoring and stimulus constancy [22,23,32]	Reflects stability of peripheral activation and stimulus delivery	Variability in M-wave indicates inconsistent stimulation	Monitor M-wave amplitude and adjust stimulus to maintain constant peripheral input
Background muscle activity (motor state) [23,26]	Modulates motoneuron excitability and reflex gain	Affects amplitude and repeatability; changes physiological interpretation	Control and report background EMG; analyze rest and contraction conditions separately
Posture and joint angle [5,32,56]	Influence muscle spindle input and afferent feedback	Alters baseline excitability and reflex amplitude	Standardize body position and joint configuration across conditions
Motor, postural, and task-related context [23,45]	Reflect integration of descending drive and sensory input	Produces task-dependent modulation of reflex amplitude	Use consistent motor/postural conditions; avoid combining different behavioral contexts
Stimulation frequency/ISI [5,7]	Engages history-dependent processes, primarily homosynaptic (post-activation) depression	Strongly influences reflex amplitude and recovery dynamics	Standardize ISI; use sufficiently long intervals (≈10 s) when baseline measurements are required
Fatigue [57,58]	Alters motoneuron excitability and motor unit behavior, with associated changes in spinal reflex transmission	Modulates H-reflex amplitude during sustained or repeated activity	Minimize fatigue, standardize task duration, and include rest periods
Normalization strategy [26,30]	Accounts for peripheral excitability differences but not all central mechanisms	Influences comparability across subjects and sessions	Normalize H-reflex amplitude to M_max_ or to a controlled reference response; interpret with caution
Muscle selection and anatomical factors [9,29,30]	Reflect differences in reflex pathway accessibility, muscle function, innervation, and segmental organization	Affects reproducibility, clinical applicability, and generalizability across muscles and limb segments	Select the target muscle according to the study question; soleus/triceps surae are common, but other limb muscles may be used; report anatomical considerations.
Averaging and repeatability [26]	Improves signal stability and reduces random variability	Insufficient repetitions increase measurement noise	Use repeated trials and averaging; number of stimuli should be adapted to the paradigm and desired reliability

M_max_, maximal M-wave; H_max_, maximal H-reflex; EMG, electromyography; ISI, interstimulus interval.

**Table 2:** Classical clinical applications of the H-reflex and their key limitations.

Domain	Parameter *	Clinical Use	Key Limitations
Conduction (proximal pathways) [9,29,45]	Latency, side-to-side comparison	S1 radiculopathy (soleus), C6–C7 pathways (FCR), proximal nerve/root lesions	Influenced by height, limb length, age; limited sensitivity in mild lesions
Conduction (diagnostic presence/absence) [5,29]	Presence/absence of H-reflex	Peripheral neuropathy, severe radiculopathy, polyneuropathy	Non-specific; may be absent in normal elderly or due to technical factors
Excitability [5,9,30]	Hmax/Mmax ratio	Spasticity, upper motor neuron syndromes, hyperreflexia	Low specificity; influenced by presynaptic inhibition, homosynaptic depression, task conditions
Segmental spinal function [5,9,23]	H-reflex amplitude, recruitment curve	Assessment of Ia afferent–motoneuron pathway integrity; spinal cord lesions; differential diagnosis (central vs. peripheral)	Strong dependence on stimulus conditions, posture, background EMG
Symmetry and comparative measures [29,45]	Interlimb latency/amplitude differences	Detection of unilateral pathology (e.g., radiculopathy)	Requires strict standardization; variability between sessions

* Interpretation of all parameters requires strict control of stimulation conditions and background muscle activity.

## Data Availability

No new data were created or analyzed in this study. Data sharing is not applicable to this article.

## Abbreviations

The following abbreviations are used in this manuscript:

AUC	Area under the curve
EMG	Electromyography
EPSPs	Excitatory postsynaptic potentials
FCR	Flexor carpi radialis
H-reflex	Hoffmann reflex
H_1_	First H-reflex response evoked in a train of stimuli
H_50_	Stimulus intensity required to elicit half-maximal H-reflex response
H_max_	Maximum amplitude of the H-reflex
H_max_/M_max_	Ratio of maximum H-reflex amplitude to maximum M-wave amplitude
HSD	Homosynaptic depression
Ia	Group Ia afferent fibers
Ib	Group Ib afferent fibers
ISI	Inter-stimulus interval
M-wave	Motor response following direct electrical activation of motor axons
M_max_	Maximum amplitude of the M-wave
RDD	Rate-dependent depression
